# Women’s Mental Health and Pregnancy Loss: What Should We Be Aware Of?

**DOI:** 10.1192/j.eurpsy.2022.2234

**Published:** 2022-09-01

**Authors:** C. Fernandes Santos

**Affiliations:** Hospital Garcia de Orta, Department Of Psychiatry And Mental Health, Almada, Portugal

**Keywords:** Pregnancy, women, pregnancy loss, perinatal mental health

## Abstract

**Introduction:**

Pregnancy loss (PL) – by induced or spontaneous termination of pregnancy – is common, although its consequences on women’s mental health are often neglected in clinical practice.

**Objectives:**

To understand the existence of psychiatric morbidity in women, associated with PL, as well as related risk factors.

**Methods:**

Non-systematic review of literature through search on PubMed/MEDLINE for publications up to 2021, following the terms ‘pregnancy loss’, ‘psychiatry disorder’, ‘depression’ and ‘anxiety’.

**Results:**

After a PL, anxiety is the most frequent symptomatology in 41% of women. Depression occurs in 22-36% of women in the first two weeks after PL, declining over time. Symptoms compatible with Post Traumatic Stress Disorder (PTSD) are found in 25% of women with PL in the first month. Women who meet criteria for PTSD are more likely to present criteria for Depressive Episode. Substance Use Disorder and Prolonged Grief Disorder are also reported, the latter having, as predictors, previous relational difficulties, poor social support and absence of descendants. Risk factors associated with significant psychopathology within PL are, for example, nulliparity, infertility, previous PL, longer gestation time, lower marital satisfaction, previous psychiatric illness, and history of abuse.

**Conclusions:**

In clinical practice, the risk of psychopathology in women with PL should be considered. This population should be actively questioned about the presence of symptoms, as well as their persistence and impact, requiring timely and appropriate pharmacological and psychotherapeutic intervention. Perinatal Mental Health interventions show important gains in the overall health of women and their offspring.

**Disclosure:**

No significant relationships.

